# Supplementary material for: Stacking Model‐Based Classifiers for Dealing With Multiple Sets of Noisy Labels
Source: Biom J. 2025 Mar 12;67(2):e70042. doi: 10.1002/bimj.70042 (PMC11898607; doi:10.1002/bimj.70042)
Supplement: Supplementary file 1 — Supporting Information [file BIMJ-67-e70042-s001.zip › density-based-ensemble-model-main/competitors_real_data/netlab-master/netlab-master/help/knn.htm]

Netlab Reference Manual knn


# knn

## Purpose

Creates a K-nearest-neighbour classifier.

## Synopsis

```
net = knn(nin, nout, k, tr_in, tr_targets)
```

## Description

`net = knn(nin, nout, k, tr_in, tr_targets)` creates a KNN model `net`
with input dimension `nin`, output dimension `nout` and `k`
neighbours. The training data is also stored in the data structure and the
targets are assumed to be using a 1-of-N coding.

The fields in `net` are

```
  type = 'knn'
  nin = number of inputs
  nout = number of outputs
  tr_in = training input data
  tr_targets = training target data
```

## See Also

`kmeans`, `knnfwd`

---

**Pages:**
Index


---

Copyright (c) Ian T Nabney (1996-9)
